# PET/CT密度比与摄取比判断肺癌纵隔淋巴结转移的研究

**DOI:** 10.3779/j.issn.1009-3419.2015.03.05

**Published:** 2015-03-20

**Authors:** 亭亭 邵, 丽娟 于, 迎辞 李, 暮楠 陈

**Affiliations:** 150081 哈尔滨，哈尔滨医科大学附属肿瘤医院PET/CT-MRI中心 Department of PET/CT-MRI, Harbin Medical University Cancer Hospital, Harbin 150081, China

**Keywords:** 正电子发射体层显像, 计算机体层成像, 密度比, 摄取比, 肺肿瘤, 淋巴结转移, Positron emission tomography, Computed tomography, Density ratio, SUV ratio, Lung neoplasms, Lymph node metastasis

## Abstract

**背景与目的:**

肺癌纵隔淋巴结转移是非常重要的生存预后因素，准确的纵隔分期可以使患者最大程度地受益于手术，正电子发射体层显像/计算机体层成像（positron emission tomography/computed tomography, PET/CT）已成为肺癌患者分期的常规手段。本研究旨在探讨18氟-氟代脱氧葡萄糖（18-fluoro-2-deoxy-glucose, ^1^8F-FDG）PET/CT在判断肺癌纵隔淋巴结转移上的价值。

**方法:**

回顾性分析72例肺癌患者术前全身PET/CT显像结果。72例患者均行根治性手术及系统纵隔淋巴结清扫，共取出413枚淋巴结，其中转移淋巴结为45枚。以病理结果作为标准，测量淋巴结短径、CT值、标准化摄取值（standardized uptake value, SUV）及纵隔血池的CT值与SUV等参数，计算淋巴结与纵隔血池密度比值以及淋巴结与纵隔血池SUV摄取比值，应用受试者工作特征（receiver operating characteristic, ROC）曲线计算截断点，分析密度比、摄取比与淋巴结良恶性关系，并与常规PET/CT法、PET/CT综合分析法比较诊断纵隔淋巴结的准确性。

**结果:**

密度比对淋巴结诊断的截断点为0.9，摄取比的截断点为1.2，当密度比≤0.9、摄取比≥1.2时，PET/CT对纵隔淋巴结诊断的准确率较高，将淋巴结短径、淋巴结最大标准化摄取值（maximum standardized uptake value, SUVmax）、密度比、摄取比综合计算PET/CT对纵隔淋巴结诊断的准确率为95.2%，而常规PET/CT法对纵隔内淋巴结诊断的准确率为89.8%，PET/CT综合分析法诊断的准确率为90.8%。

**结论:**

将PET/CT密度比、摄取比与淋巴结短径及SUVmax综合在一起对纵隔淋巴结诊断的准确率较高，优于常规PET/CT法及PET/CT综合分析法。

## Abstract (en)

**Background and objective:**

Mediastinal involvement in lung cancer is a highly significant prognostic factor for survival, and accurate staging of the mediastinum will correctly identify patients who will benefit the most from surgery. Positron emission tomography/computed tomography (PET/CT) has become the standard imaging modality for the staging of patients with lung cancer. The aim of this study is to investigate 18-fluoro-2-deoxy-glucose (^18^F-FDG) PET/CT imaging in the detection of mediastinal disease in lung cancer.

**Methods:**

A total of 72 patients newly diagnosed with non-small cell lung cancer (NSCLC) who underwent preoperative whole-body ^18^F-FDG PET/CT were retrospectively included. All patients underwent radical surgery and mediastinal lymph node dissection. Mediastinal disease was histologically confirmed in 45 of 413 lymph nodes. PET/CT doctors analyzed patients' visual images and evaluated lymph node's short axis, lymph node's maximum standardized uptake value (SUVmax), node/aorta density ratio, node/aorta SUV ratio, and other parameters using the histopathological results as the reference standard. The optimal cutoff value for each ratio was determined by receiver operator characteristic curve analysis.

**Results:**

Using a threshold of 0.9 for density ratio and 1.2 for SUV ratio yielded high accuracy for the detection of mediastinal disease. The lymph node's short axis, lymph node's SUVmax, density ratio, and SUV ratio of integrated PET/CT for the accuracy of diagnosing mediastinal lymph node was 95.2%. The diagnostic accuracy of mediastinal lymph node with conventional PET/CT was 89.8%, whereas that of PET/CT comprehensive analysis was 90.8%.

**Conclusion:**

Node/aorta density ratio and SUV ratio may be complimentary to conventional visual interpretation and SUVmax measurement. The use of lymph node's short axis, lymph node's SUVmax, and both ratios in combination is better than either conventional PET/CT analysis or PET/CT comprehensive analysis in the assessment of mediastinal disease in NSCLC patients.

尽管诊断和治疗策略不断更新，肺癌仍然是全球癌症致死的首要原因，其中80%为非小细胞肺癌（non-small cell lung cancer, NSCLC）^[1]^。NSCLC纵隔淋巴结转移是非常重要的生存预后因素，准确的纵隔淋巴结分期能使患者最大程度地受益于手术，约21%-50%新增NSCLC病例存在淋巴结转移。目前，18氟-氟代脱氧葡萄糖正电子发射体层显像/计算机体层成像（18-fluoro-2-deoxy-glucose positron emission tomography/computed tomography, ^18^F-FDG PET/CT）已经广泛应用于肺癌的诊断、分期、预后及治疗效果评估^[2, 3]^，虽然文献^[4-6]^报道PET/CT诊断纵隔淋巴结转移的敏感性为69%-80%，特异性为82%-94%，但是如何可以进一步地提高PET/CT肺癌纵隔淋巴结转移诊断的准确性，成为国内外学者研究的重点。本研究回顾性分析我院术前行PET/CT检查并经手术病理证实72例NSCLC患者的资料，探讨PET/CT诊断NSCLC患者纵隔淋巴结转移的临床应用价值。

## 1 资料与方法

### 1.1 一般资料

收集自2011年2月-2013年10月期间在我院术前接受^18^F-FDG PET/CT检查的NSCLC患者72例，所有患者均在行PET/CT检查后两周时间内行肺癌根治手术及系统纵隔淋巴结清扫，且PET/CT检查前所有患者均未行任何放疗或化疗等干预性治疗。72例患者中，男性44例，女性28例。年龄39岁-78岁，平均（61±8.01）岁。肺癌病理类型：腺癌52例，鳞癌15例，腺鳞癌4例，肉瘤样癌1例。

### 1.2 PET/CT扫描

采用美国通用电器医疗仪器公司生产的PET/CT显像仪（discovery elite, GE）。显像剂^18^F-FDG由回旋加速器和化学合成系统自动合成，放化纯度＞95%。所有患者检查前均需空腹6 h以上，测量身高及体重，检查前将空腹血糖浓度控制在7 mmol/L以内。经肘静脉注射^18^F-FDG，按5.55 MBq/kg-7.40 MBq/kg，注射显像剂后嘱患者安静休息60 min后进行扫描。患者先行64排全身螺旋CT扫描（从颅顶到双侧股骨），管电压140 KV，管电流150 mA，层厚3.75 mm，矩阵512×512，球管单圈旋转时间0.8 s。随后行PET扫描，每个床位采集2.5 min-3 min，一般采集6个-7个床位。图像重建采用有序子集最大期望值法（ordered subsets expectation maximization, OSEM），利用CT投射扫描数据对PET图像进行衰减校正。图像融合通过工作站的软件进行，同时获得横断面、矢状面和冠状面的PET、CT及两者融合图像。

### 1.3 图像分析

72例患者均于PET/CT检查后的两周内行肺癌根治性手术及系统纵隔淋巴结清扫，参照美国胸科学会（American Thoracic Society, ATS）制订的纵隔淋巴结分区标准^[7]^，手术医师将所切除的各组淋巴结编号、记录后送病理检查，然后由两位有5年以上影像诊断经验的PET/CT医师在不知道病理结果的情况下，分别对PET/CT图像进行分析，有分歧时共同商讨得出一致意见。同样按照ATS纵隔淋巴结分区标准记录每一个淋巴结位置，分别测量各淋巴结的SUVmax、淋巴结短径、CT值以及肺癌原发灶的SUVmax。应用视觉分析法比较每个淋巴结与纵隔血池CT纵隔窗上的密度差异，同样比较每个淋巴结与纵隔血池PET图像上浓聚程度的差异。然后再测量纵隔血池CT密度与PET浓聚程度，测量方法为：在纵隔血池（即主动脉弓层面）上画一个感兴趣区（region of interest, ROI）^[8]^，测量此区域的CT值、平均标准化摄取值（mean standardized uptake value, SUVmean）并记录。常规PET/CT对纵隔内淋巴结的诊断标准为：CT图像上淋巴结短径≥1 cm，且PET图像上淋巴结SUVmax≥2.5者为阳性。将常规PET/CT与视觉分析综合在一起（即PET/CT综合分析法）对纵隔内淋巴结的诊断标准为：淋巴结短径≥1 cm、淋巴结SUVmax≥2.5、视觉分析淋巴结密度等于或低于纵隔血池密度、视觉分析淋巴结放射性浓聚程度高于纵隔血池者为阳性^[9]^。

### 1.4 统计学方法

使用SPSS 19.0统计软件进行数据分析，计数资料组间比较使用χ^2^检验，计量资料采用独立样本*t*检验，ROC曲线用来评估变量的敏感性和特异性，以*P*＜0.05为差异有统计学意义。

## 2 结果

### 2.1 全部淋巴结检查结果

72例NSCLC患者经系统纵隔淋巴结清扫术，共清扫出413枚淋巴结，淋巴结分布无明显规律，54.7%（226/413）分布在4组和7组，其中7组129枚，4组97枚。每枚淋巴结均经病理检查，可见癌细胞者为转移性淋巴结，否则为非转移性淋巴结，413枚淋巴结中转移性淋巴结为45枚（4组13枚，7组13枚，2组9枚，10组7组，5组2枚，3组1枚），非转移性淋巴结368枚（表 1）。

**1:** 转移性与非转移性淋巴结各参数列表 Comparison of different parameters of metastatic and non-metastatic lymph nodes

Pathology group	Number of nodes	Short axis of nodes (cm)	SUVmax of nodes	SUVmax of primary tumors
Metastatic lymph nodes	45	0.97±0.30	3.92±1.88	11.18±4.93
Non-metastatic lymph nodes	368	0.62±0.23	2.31±1.23	10.79±5.45

应用独立样本*t*检验对转移性与非转移性淋巴结短径、SUVmax以及淋巴结所对应的原发灶的SUVmax进行统计学分析，统计学结果显示淋巴结转移组和非转移组，两组淋巴结平均短径、平均SUVmax间具有统计学差异（*t*=-7.49, *P*＜0.001; *t*=-5.59, *P*＜0.001）；转移与非转移组淋巴结所对应的肺原发灶的平均SUVmax之间不存在统计学差异（*t*=-0.461, *P*=0.65）。

### 2.2 淋巴结与纵隔血池的密度比和摄取比

计算淋巴结与纵隔血池密度比值以及淋巴结与纵隔血池SUV摄取比值，应用ROC曲线计算截断点。密度比对淋巴结诊断的截断点为0.9，曲线下面积为0.755（图 1），以0.9为诊断淋巴结良恶性的截断点，密度比≤0.9记为转移性淋巴结，计算得出以0.9作为截断点对淋巴结良恶性诊断的敏感性为68.9%，特异性为79.9%；摄取比的截断点为1.2，曲线下面积为0.780（图 2），以1.2为诊断淋巴结良恶性的截断点，摄取比≥1.2记为转移性淋巴结，计算得出以1.2作为截断点对淋巴结良恶性诊断的敏感性为86.7%，特异性为60.6%。我们将密度比与摄取比相互结合，当密度比≤0.9、摄取比≥1.2时诊断为恶性淋巴结，将两者结合起来对纵隔淋巴结良恶性的诊断明显高于单独诊断，敏感性为82.2%，特异性为83.2%（表 2）。

**1:**
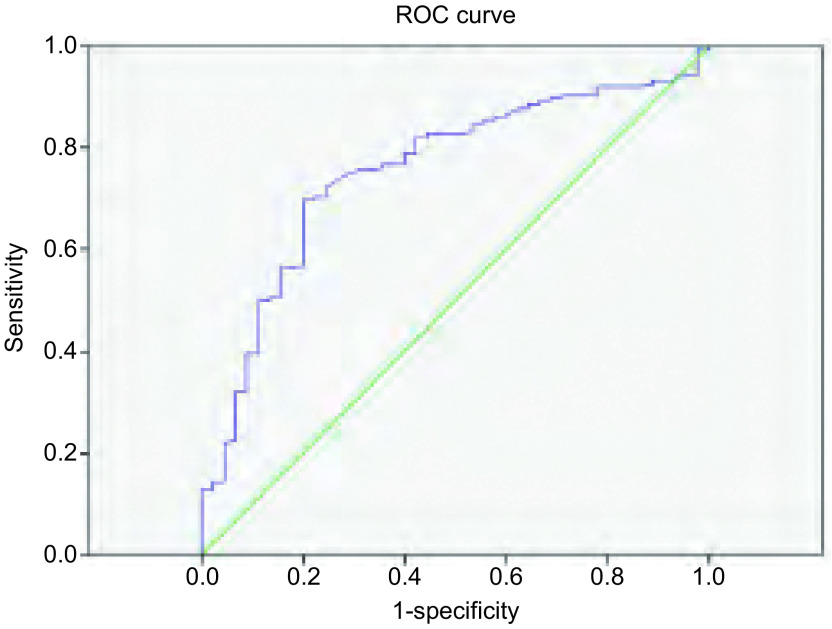
淋巴结与纵隔血池密度比的ROC曲线 ROC curve for node/aorta density ratio, with AUC of 0.755. AUC: area under the cure; ROC: receiver operating characteristic curve.

**2:**
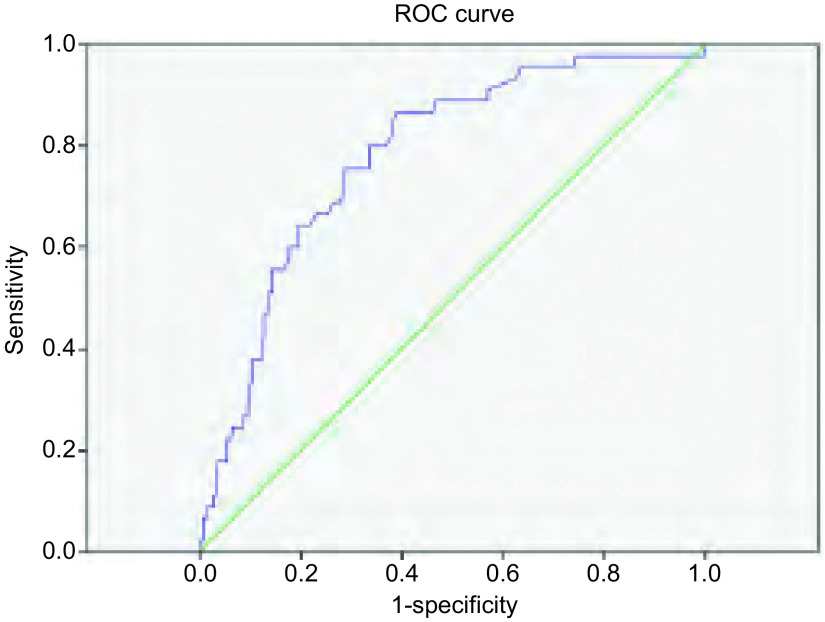
淋巴结与纵隔血池摄取比的ROC曲线 ROC curve for node/aorta SUV ratio, with AUC of 0.780

**2:** 不同比值的诊断效能 The diagnostic efficacy of different ratios

Imaging criteria	Sensitivity	Specificity
Node/aorta density ratio 0.9	68.9% (31/45)	79.9% (294/368)
Node/aorta SUV ratio≥1.2	86.7% (39/45)	60.6% (223/368)
Node/aorta (density ratio≤0.9 + SUV ratio≥1.2)	82.2% (37/45)	83.2% (306/368)

### 2.3 淋巴结各种PET/CT诊断方法的比较

将413枚淋巴结PET/CT诊断结果与病理结果进行对照，分别计算常规PET/CT法、PET/CT综合分析法、PET/CT综合分析法结合两个比值（即同时满足淋巴结短径≥1 cm、淋巴结SUVmax≥2.5、视觉分析淋巴结密度等于或低于纵隔血池密度且测量密度比≤0.9、视觉分析淋巴结放射性浓聚程度高于纵隔血池且测量摄取比≥1.2者为阳性）对纵隔淋巴结诊断的敏感性、特异性、阳性预测值、阴性预测值及诊断准确率（表 3）。PET/CT综合分析法结合两个比值对纵隔淋巴结诊断的准确率较高，优于常规PET/CT法及PET/CT综合分析法，差异有统计学意义（χ^2^=5.4, *P*＜0.05）。

**3:** 不同PET/CT诊断方法的效能比较 The diagnostic efficacy of different PET/CT diagnostic methods.

PET/CT diagnosis method	Pathology		Sensitivity (%)	Specificity (%)	Positive predictive value (%)	Negative predictive value (%)	Diagnosis rate (%)
	Malignant	Benign					
Convention			71.1	92.1	51.6	96.3	89.8
Malignant	32	29					
Benign	13	339					
Comprehensive analysis			51.1	95.7	58.9	94.1	90.8
Malignant	23	16					
Benign	22	352					
Comprehensive analysis+two ratios			68.9	98.4	83.4	96.2	95.2
Malignant	31	6					
Benign	14	362					

### 2.4 误诊淋巴结检查结果

将两个比值与PET/CT综合分析法结合对纵隔淋巴结进行分析发现本研究413枚淋巴结中仅有6枚淋巴结（假阳性）被误诊为恶性淋巴结（图 3），这6枚假阳性淋巴结来源于4例肺癌患者，均合并肺炎、肺气肿等肺部良性疾病，但无结核感染史。9例肺癌患者的14枚转移淋巴结被误诊为良性，这14枚假阴性淋巴结的体积均较小（0.63±0.14）cm，明显低于本研究全部恶性淋巴结体积的平均水平（1.0±0.30）cm。

**3:**
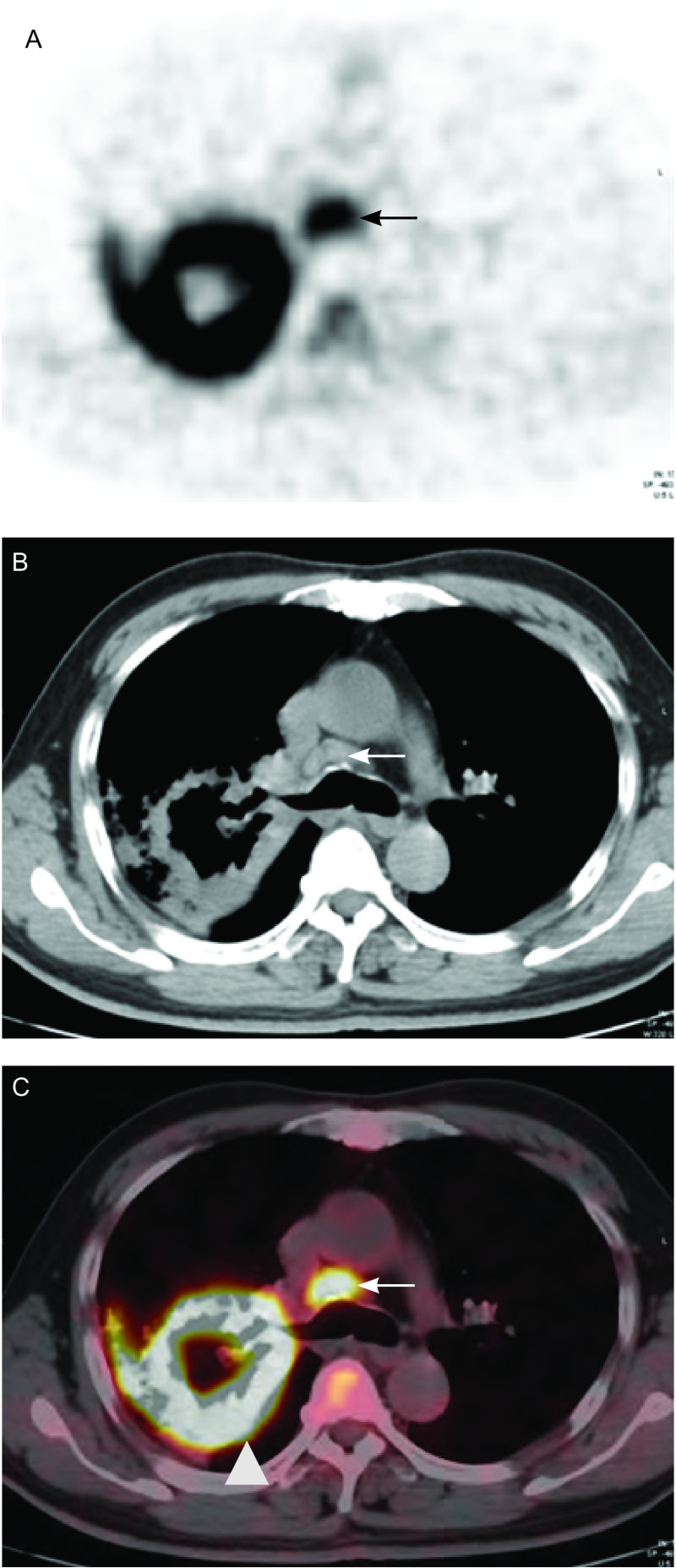
男性患者，60岁，右肺上叶鳞癌。PET示纵隔内异常高代谢灶（A），CT示纵隔内（4组）淋巴结短径为1.1 cm（B），PET/CT示纵隔内淋巴结SUVmax为4.7，原发灶SUVmax为9.2（C）。手术病理证实此枚淋巴结为良性淋巴结。 A 60-year-old man with squamous cell carcinoma in right upper lobe. The PET (A) image showed the abnormal uptake in the mediastinum (arrow). Mediastinal-window view of transverse CT (B) scan showed lymph node with short axis of 1.1 cm in 4R group (arrow). PET/CT (C) showed increased ^18^F-FDG uptake of mediastinal lymph node (arrow) and primary tumor (arrowhead) (SUVmax=4.7 and 9.2, respectively). This lymph node was negative for metastasis on the pathological examination. PET-CT: positron emission tomography/computed tomography.

## 3 讨论

据世界卫生组织统计，肺癌已经成为全球第一癌症杀手，目前临床对肺癌的治疗手段仍以手术为主，由于肺癌较容易发生淋巴结转移，因此准确的纵隔分期可以使患者避免不必要的开胸手术，减少手术造成的损伤，同时防止患者错失有效手术机会^[9]^。因为存在较高特异性和阴性预测值，PET/CT已成为NSCLC患者分期的常规手段。

研究表明，^18^F-FDG PET/CT在检测NSCLC患者纵隔分期和远处转移方面优于其他成像方式，然而仅凭借传统视觉分析PET/CT不足以完全排除假阴性结果，并且由于炎症等良性病变存在可以引起淋巴结FDG聚集而呈假阳性表现，常规PET/CT法可能会高估或者低估NSCLC患者的纵隔分期。我们研究发现将PET/CT测量法以及视觉分析法相互结合的PET/CT综合分析法可明显提高PET/CT对纵隔淋巴结诊断的准确率，国内外有研究^[8, 10]^提出淋巴结密度比与摄取比对淋巴结良恶性诊断有意义。因此本研究同时引入这两个新的参数，即淋巴结与纵隔血池密度比以及摄取比，应用ROC曲线分析得出，当密度比≤0.9、摄取比≥1.2时，PET/CT对纵隔淋巴结诊断的准确率较高，将两个比值与PET/CT综合分析法结合计算PET/CT对纵隔淋巴结诊断的敏感性、特异性、阳性预测值、阴性预测值及准确率分别为68.9%、98.4%、83.4%、96.2%、95.2%，其诊断准确率明显高于常规PET/CT法及PET/CT综合分析法，差异有统计学意义（*P*＜0.05）。

本研究共分析了413枚淋巴结，其中恶性淋巴结45枚，良性淋巴结368枚。将两个比值与常规PET/CT法结合对纵隔淋巴结进行分析，仅有6枚良性淋巴结被误诊为恶性（来源于4例肺癌患者），14枚恶性淋巴结被误诊为良性（来源于9例肺癌患者）。相关文献^[11-13]^报道，假阳性病例主要因为炎症和肉芽肿性病变。本研究中4例假阳性病例皆合并肺炎、肺气肿等肺部良性疾病，不存在结核感染史，分析6枚假阳性淋巴结可能与炎症有关。奥斯等认为假阴性结果在原发灶SUVmax更低和淋巴结更小的病例中比较常见^[14]^，而本研究中良恶性两组淋巴结原发灶SUVmax分别为（10.8±5.45）及（11.2±4.93），差异无统计学意义（*P*＞0.05），两组淋巴结短径分别为（0.6±0.23）cm及（1.0±0.30）cm，差异有统计学意义（*P*＜0.05），分析假阴性结果主要原因可能在于淋巴结短径较小，假阴性淋巴结短径均小于1.0 cm，最小者仅为0.4 cm。此外，部分容积效应也会影响小淋巴结的放射性摄取^[11]^。

本研究的局限性在于72例患者的413枚淋巴结，仅有45枚转移淋巴结，结果可能存在一定偏移。此外，未知的患者因素也可能影响结果，今后需依据较大的样本量以方便进一步研究。

综上所述，将淋巴结与纵隔血池的密度比及摄取比与PET/CT综合分析法相互结合可以提高PET/CT对NSCLC患者纵隔分期的准确率，优于常规PET/CT法及PET/CT综合分析法。
